# Comparative Transcriptional Analysis of Asexual and Sexual Morphs Reveals Possible Mechanisms in Reproductive Polyphenism of the Cotton Aphid

**DOI:** 10.1371/journal.pone.0099506

**Published:** 2014-06-10

**Authors:** Li-Jun Liu, Hong-Yuan Zheng, Feng Jiang, Wei Guo, Shu-Tang Zhou

**Affiliations:** 1 State Key Laboratory of Integrated Management of Pest Insects and Rodents, Institute of Zoology, Chinese Academy of Sciences, Beijing, China; 2 Beijing Institutes of Life Science, Chinese Academy of Sciences, Beijing, China; Kansas State University, United States of America

## Abstract

Aphids, the destructive insect pests in the agriculture, horticulture and forestry, are capable of reproducing asexually and sexually upon environmental change. However, the molecular basis of aphid reproductive mode switch remains an enigma. Here we report a comparative analysis of differential gene expression profiling among parthenogenetic females, gynoparae and sexual females of the cotton aphid *Aphis gossypii*, using the RNA-seq approach with next-generation sequencing platforms, followed by RT-qPCR. At the cutoff criteria of fold change ≥2 and *P*<0.01, we identified 741 up- and 879 down-regulated genes in gynoparae versus parthenogenetic females, 2,101 up- and 2,210 down-regulated genes in sexual females compared to gynoparae, and 1,614 up- and 2,238 down-regulated genes in sexual females relative to parthenogenetic females. Gene ontology category and KEGG pathway analysis suggest the involvement of differentially expressed genes in multiple cellular signaling pathways into the reproductive mode transition, including phototransduction, cuticle composition, progesterone-mediated oocyte maturation and endocrine regulation. This study forms a basis for deciphering the molecular mechanisms underlying the shift from asexual to sexual reproduction in the cotton aphid. It also provides valuable resources for future studies on this host-alternating aphid species, and the insight into the understanding of reproductive mode plasticity in different aphid species.

## Introduction

Reproductive polyphenism, the capability of reproducing asexually and sexually upon environmental changes is a typical phenotypic plasticity in insects such as the aphids [1]–[3]. Aphids have about 4,400 species belonging to the superfamily of Aphidoidea in the order of Hemiptera, and are among the most destructive insect pests in the agriculture, horticulture and forestry. Their large population and wide distribution are due to their reproductive polyphenism. During spring and summer, aphids undergo several generations of asexual reproduction via viviparous parthenogenesis. In the fall, however, aphids are able to produce oviparous females and males to lay overwintering eggs. Although the reproductive polyphenism of aphids has been known for decades, the molecular basis of transition between asexual and sexual reproduction is not uncovered. Attempts have been made to detect genes in response to shortening photoperiod in the pea aphid *Acyrthosiphon pisum*, a non-host-alternating aphid species [4], [5]. By cDNA microarray analysis, 33 up-regulated (1.3 to 2.5 fold) and 26 down-regulated (−1.4 to −3.2 fold) transcripts are identified in the pea aphids reared under the 12L:12D photoperiod compared to 16L:8D, which are confirmed by quantitative reverse transcriptase PCR (RT-qPCR) on 2 cuticle (*CuPr1*, *CuPr2*) and 3 signaling (*HDD11*, *Wunen*, and *Dreg-5*) genes [6]. When pea aphids are reared under the 10L:14D photoperiod, transcript levels of genes coding for a tubulin binding protein (*Tub56D*), a cuticle protein (*Cutic-4*) and a non-annotated protein (*No-hit-1*) are significantly increased, whereas mRNA levels of genes coding for another cuticle protein (*Cutic-5*), a glutamate-ammonia ligase (*Gs2*) and a protein with nitrilase activity (*CG8132*) are significantly decreased [7]. Comparison of pea aphids reared outdoor in June and September via microarray analysis demonstrates 367 differentially expressed transcripts [8]. In another microarray analysis, 33 genes are found to be differentially expressed in asexual versus sexual embryos of pea aphids maintained at 12L:12D [9].

Despite the studies on reproductive polyphenism of the pea aphid using the microarray-based technology, the genetic information of aphid reproductive mode plasticity remains limited, and a large-scale analysis of differentially expressed genes in reproductive morphs of a host-alternating aphid species like the cotton aphid, *Aphis gossypii* has been lacking. The cotton aphid, also known as the melon aphid is a worldwide agricultural insect pest causing damage by phloem sap-sucking, transmission of plant virus, and production of honeydew contamination on cotton [10]. The holocyclic life cycle of cotton aphids is characterized by the multiple reproductive morphs including the parthenogenetic female, gynopara and sexual female (Fig. S1). Parthenogenetic females reproduce in parthenogenesis, which allows rapid population growth on preferred host plants such as the cotton and melon in late spring and summer. In addition to host plant alternating, the cotton aphid differs from the pea aphid with the existence of gynoparae that are produced by parthenogenetic females in the fall. Gynoparae are alate, and migrate to the primary host plants such as the prickly ash and althea tree to produce sexual females that mate with alate males and lay fertilized eggs for overwintering [11]. Although the transition from asexual to sexual reproduction is clearly evident for the holocyclic life cycle of cotton aphids, the underlying molecular mechanisms have not been explored.

Recently, RNA-seq approach with the next-generation sequencing platform has offered a high-throughput methodology and in-depth analysis of gene expression profiling [12]–[14]. Compared to the microarray-based technologies, RNA-seq detects more transcripts and yields better quantity of gene expression because of its higher sensitivity for detection of low abundant transcript and comprehensive information for unannotated transcripts [12], [13]. In this study, we report the identification of differentially expressed gene (DEG) profiling among the parthenogenetic female, gynopara and sexual female of cotton aphids using the RNA-seq approach, followed by RT-qPCR. The putative roles of selected DEGs and significantly enriched signal pathways potentially involved in the transition from viviparous parthenogenesis and sexual reproduction were discussed. This study provides the most comprehensive dataset to date for gene expression in reproductive morphs of the cotton aphid, which could facilitate our understanding of molecular mechanisms in reproductive mode switch of aphids.

## Materials and Methods

### Animals

**Figure 1:**
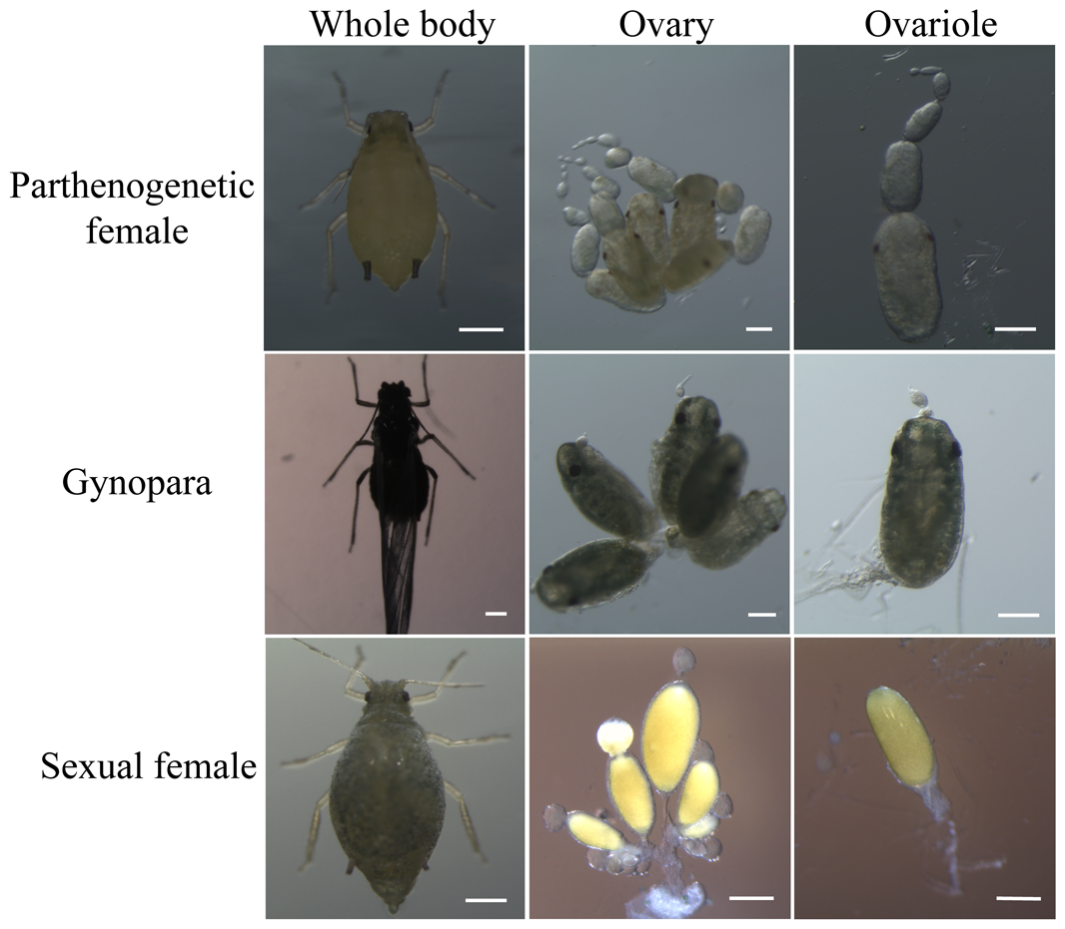
Body, ovarian and ovariole morphology of three reproductive morphs of the cotton aphid. Both the parthenogenetic female and gynopara reproduce in parthenogenesis, but their body, ovary and ovarioles are discriminated. The sexual female has divergent morphology from other two morphs and produces eggs. Scale bars: body, 0.2 mm; ovary and ovariole, 0.1 mm.

Cotton aphids were reared on cotton plants under the photoperiod of 16L:8D and at 25°C for parthenogenetic reproduction. Aphid population density and cotton plant growth were monitored routinely to avoid the production of alate parthenogenetic aphids. To induce the production of gynoparae and oviparous sexual females, 2-day-old apterous parthenogenetic female adults were transferred to separate cotton plants and maintained under the photoperiod of 8L:16D and at 18°C. Gynoparae emerged at the second generation. Subsequently, gynoparae were transferred individually to separate cotton plants (one aphid per isolated plant) under the photoperiod of 8L:16D and at 18°C to produce sexual females. These three reproductive morphs have distinct morphological characteristics as shown in Fig. 1. The 2-day-old adult aphids were collected individually and 30 adults of each reproductive morph were pooled. Aphids collected from different cages were used for biological replications. All the samples were immediately frozen in liquid nitrogen and stored at −80°C.

### RNA-seq library construction

The RNA-seq approach was employed to analyze the gene expression profiling among reproductive morphs [12]. Considering that the cotton aphid bears the traits of reproductive mode plasticity in multiple tissues and that the adult body size is about 1.0–1.5 mm in length, total RNA was extracted using Trizol reagent (Invitrogen) from the whole body of 2-day-old adult aphids except the gynoparae, from which embryos were removed to eliminate the potential influence by the sexual offspring. RNA purity and integrity were analyzed using Agilent 2100 Bioanalyzer. mRNA was enriched from DNase I-treated total RNA using the oligo (dT) magnetic beads, and cDNA was reverse-transcribed using the random hexamer primer. After purification with magnetic beads, cDNA was ligated at 3′-end with adenine and sequencing adaptors, followed by PCR amplification. A total of 9 libraries were constructed, including 3 replicates of parthenogenetic females, gynoparae and sexual females. The quality and quantity of all libraries were confirmed by Agilent 2100 Bioanaylzer and ABI StepOnePlus Real-Time PCR System.

### Gene profiling and data processing

All libraries were sequenced in single read modes on an Illumina HiSeq 2000 platform at Beijing Genomics Institute. After sequencing, clean reads were obtained by filtering out the adapter sequences and low-quality reads from raw reads and mapped to the cotton aphid transcriptome database (sequenced from the mixed samples of parthenogenetic females, gynoparae, sexual females and sexual males; NCBI SRA accession#, SRR1251991) by the SOAP2 program [15]. The relative expression levels of all the matched unigenes were normalized by transforming the clean data to reads per kilobases of transcripts per million mapped reads (RPKM) as described previously [14], [16]. Relative gene expression levels in each reproductive morph were calculated by average RPKM of 3 replicates. *P* values in multiple tests were corrected by false discovery rate (FDR) [17]. Fold change (FC) ≥2 and *P*<0.01 were used to determine differentially expressed transcripts. The unigenes were annotated against the NCBI Nr, Swiss-Prot, Kyoto Encyclopedia of Genes and Genomes (KEGG) and Orthologous Groups (COG) of protein database by BLASTX and with the cutoff E-value of 1E^−5^. Gene ontology (GO) function categories were determined using the Blast2GO suites and GO enrichment was performed by Web Gene Ontology Annotation Plot (WEGO, http://wego.genomics.org.cn/cgi-bin/wego/index.pl) [18]. The observed number of differentially expressed transcripts in each GO category was compared with the corresponding number of unigenes to assess the significant over-representation of differentially expressed transcripts in GO categories. Statistical significance of over-representation for each GO category was determined by Pearson Chi-Square test at *P*<0.05. Significantly enriched KEGG pathways were identified by KEGG Orthology-Based Annotation System (KOBAS 2.0) at the cutoff criteria of *P*<0.05 in hypergeometric tests [19].

### RT-qPCR

Total RNA was extracted as described above, and cDNA was reverse-transcribed from total RNA using FastQuant cDNA kit (Tiangen) following the manufacture' manual. RT-qPCR was performed using Mx3005P detection system (Agilent) at 95°C for 2 min, and then 40 cycles at 95°C for 20 s followed by 58°C for 20 s and 68°C for 20 s with ribosomal protein L27 gene (*rpl27*) as the endogenous control. cDNA equivalent to 20 ng of total RNA, 0.5 µM primer pairs and SYBR Green Real Master Mix (Tiangen) were used in each reaction. The 2^−ΔΔCt^ method was applied to analyze relative gene expression levels [20]. Primers used in RT-qPCR were given in Table S1.

## Results and Discussion

### Identification of differentially expressed genes among three reproductive morphs

**Figure 2:**
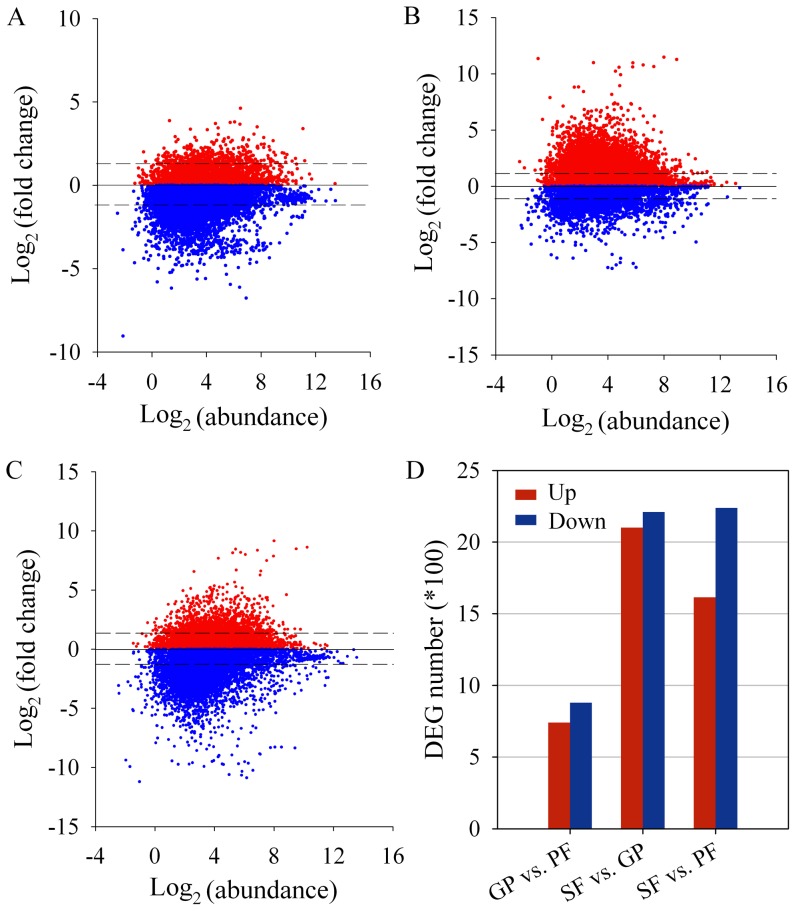
Relative gene expression profiles in the pair-wise comparison of three reproductive morphs. (A–C) Distribution of annotated unigenes in gynoparae vs. parthenogenetic females (A), sexual females vs. gynoparae (B), and sexual females vs. parthenogenetic females (C). Log_2_ transformed fold change was plotted against log_2_ transformed RPKM (abundance). Red dots, up-regulated unigenes; blue dots, down-regulated unigenes; dash lines, log_2_ (fold change)  = +1 or −1; solid line, log_2_ (fold change)  = 0. (D) Number of differentially expressed genes (DEGs) at log_2_ (fold change) ≥ +1 or ≤−1 (i.e., fold change ≥2) and *P*<0.01 in the pair-wise comparison of three reproductive modes. PF, parthenogenetic females; GP, gynoparae; SF, sexual females; Up, up-regulated DEGs; Down, down-regulated DEGs.

The parthenogenetic female, gynopara and sexual female of cotton aphids have divergent morphology in bodies, ovaries and ovarioles (Fig. 1). From the RNA-seq libraries, the total number of clean reads per library ranged from 11.5 to 12.1 million for parthenogenetic females, from 10.1 to 12.1 million for gynoparae, and from 11.5 to 12.0 million for sexual females, respectively (Fig. S2). The RNA-seq datasets of these three reproductive morphs are available at the NCBI SRA under the accession number SRR1257338, SRR1257339, and SRR1257340. After mapped to the cotton aphid transcriptome, 7.8–8.5, 6.9–7.9 and 7.7–8.0 million unique clean reads which covered 39,359–39,440, 35,158–36,941, and 33,356–37,513 unigene sequences were identified for parthenogenetic females, gynoparae and sexual females, respectively (Fig. S2). Similar numbers of clean reads and unigene sequences in the 3 libraries of each reproductive morph suggest that little bias exists in the construction of 9 libraries. Pearson correlation coefficient analysis by SPSS 19.0 software indicated a significant positive correlation (*P*<0.0001) among the 3 libraries of each reproductive morph (Table S2). The merged datasets yielded a total of 44,310 genes, which were either ubiquitously expressed in all three morphs or specifically expressed in one or two morphs (Fig. S2). After filtering out the repetitive genes and genes without annotation, 11,350 unigenes were obtained and subjected to further analysis (Fig. S2). Fold changes of unigene transcripts in the pair-wise comparison of three reproductive morphs plotted against their abundance revealed a large number of genes at variable abundance (Fig. 2A–C). Based on the cutoff criteria (FC ≥2, *P*<0.01), 1,620 DEGs including 741 up-regulated (FC  = 2.0–6078.1) and 879 down-regulated (FC  = 3.5–619.6) genes were identified in gynoparae compared to parthenogenetic females (Fig. 2D). For sexual females vs. gynoparae, 4,311 DEGs were found, including 2,101 up-regulated (FC  = 2.1–2871.8) and 2,210 down-regulated (FC  = 2.0–1891.4) genes (Fig. 2D).When sexual females were compared to parthenogenetic females, 1,614 up-regulated (FC  = 3.2–11337.6) and 2,238 down-regulated genes (FC  = 2.0–2598.9) were detected (Fig. 2D). The complete list of DEGs in the pair-wise comparison of three reproductive morphs was given in Table S3.

Since both gynoparae and parthenogenetic females undergo parthenogenesis, the divergence of gene expression between these two morphs may primarily contribute to the difference in body color, cuticle, wing and ovarian development. A remarkably increased number of DEGs between sexual females and gynoparae and between sexual females and parthenogenetic females indicate that a large set of genes is potentially involved in the transition between asexual and sexual reproduction. In this study, we detected considerably more DEGs in the cotton aphid compared to the previous studies on pea aphids [6]–[8]. This is possibly due to more distinct traits among the parthenogenetic female, gynopara and sexual female of the cotton aphid. In contrast, the oviparous and viviparous adults of the pea aphid are almost morphologically identical. Moreover, compared to cDNA microarray, RNA-seq is more sensitive, which allows the detection of more transcripts [12], [13]. The large numbers and diverse functions of DEGs may also reflect the complexity in the regulation of reproductive mode switch of the cotton aphid.

### GO category and enrichment analysis of differentially expressed genes

To uncover the functions of DEGs among the three reproductive morphs, we initially analyzed GO terms with pooled up- and down-regulated DEGs. For gynoparae vs. parthenogenetic females, 1,439 DEGs were clustered into the category of biological process, 634 DEGs into molecular function and 486 DEGs into cellular component, respectively (Fig. S3). Between sexual females and gynoparae, 3,829 DEGs were assigned to biological process, 1,678 DEGs to molecular function and 1,466 DEGs to cellular component (Fig. S3). In the comparison of sexual females and parthenogenetic females, 3,281 DEGs were categorized into biological process, 1,428 DEGs into molecular function and 1,110 DEGs into cellular component (Fig. S3).

**Figure 3:**
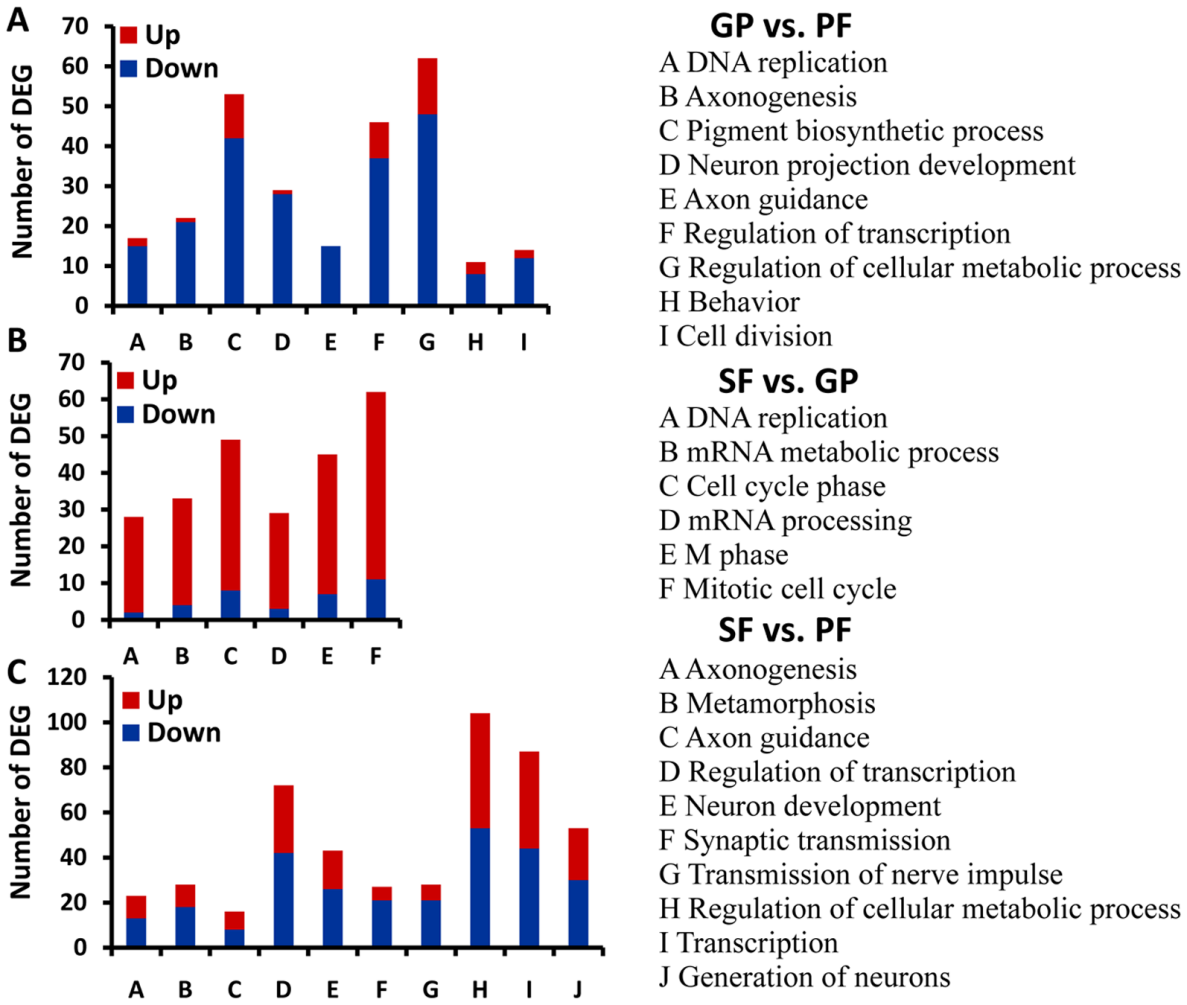
Significantly enriched GO terms of DEGs in the category of biological process. (A) Represented GO terms and the number of DEGs in gynoparae (GP) vs. parthenogenetic females (PF). (B) Represented GO terms and the number of DEGs in sexual females (SF) vs. GP. (C) Represented GO terms and the number of DEGs in SF vs. PF. Up, up-regulated; Down, down-regulated. The term was considered to be enriched significantly when the *P* values of Pearson Chi-Square test were <0.05.

To further unveil the functions of DEGs in the pair-wise comparisons, we performed the multilevel GO enrichment analysis (see Table S4 for the list of significantly enriched GO terms). In the category of biological process, the most represented GO terms of gynoparae vs. parthenogenetic females were related to DNA replication and cell division, neuron development and signaling (axonogenesis, neuron projection development, and axon guidance), pigment biosynthesis, behavior, regulation of transcription, and regulation of cellular metabolic process (Fig. 3A). For sexual females vs. gynoparae, DEGs were primarily termed to DNA replication, cell cycle, mRNA processing and metabolic process (Fig. 3B). DEGs of sexual females vs. parthenogenetic females were predominantly categorized into neuron development and signaling (axonogenesis, neuron development, axon guidance, synaptic transmission, transmission of nerve impulse, and generation of neurons), metamorphosis, transcription, and cellular metabolic process (Fig. 3C).

**Figure 4:**
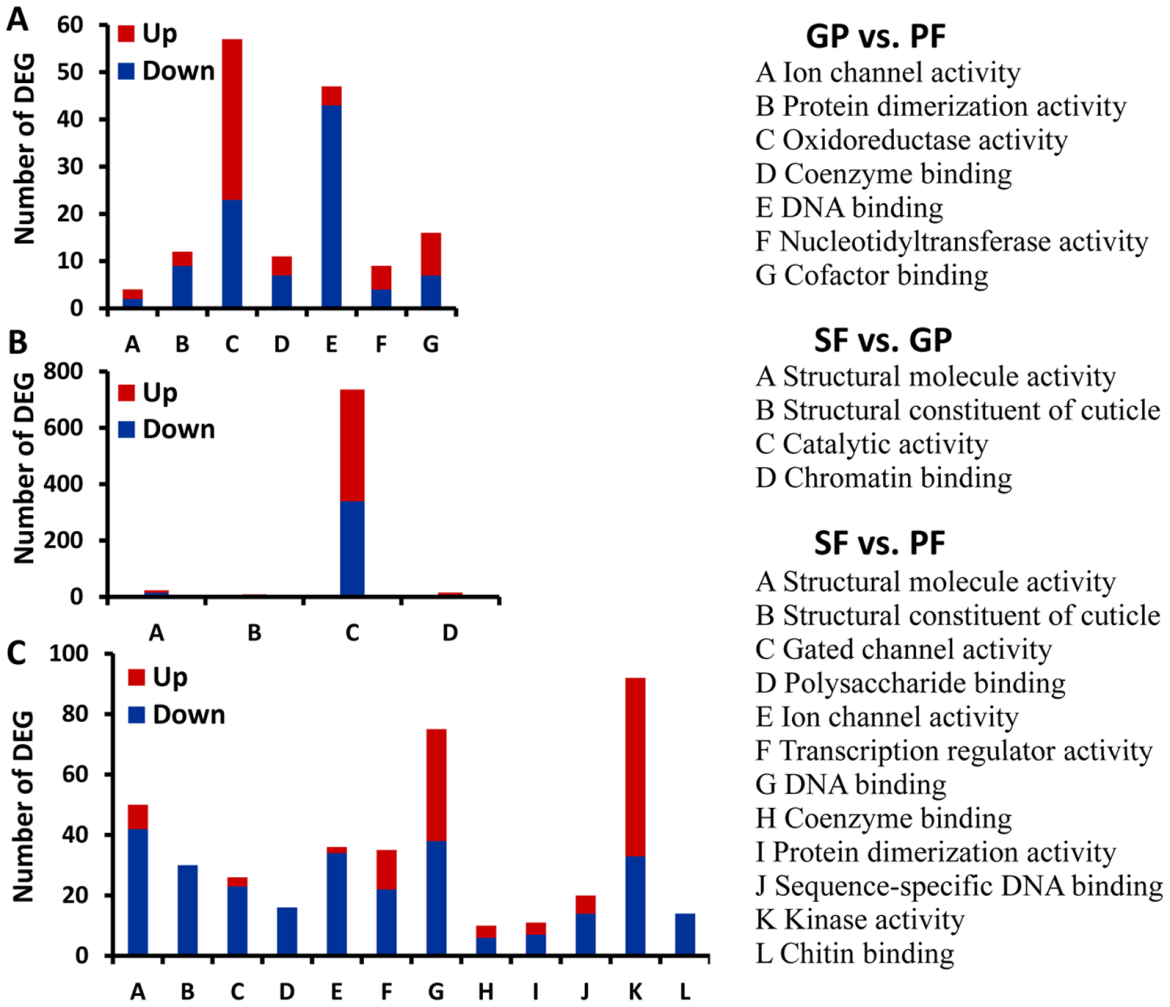
Significantly enriched GO terms of DEGs in the category of molecular function. (A) GO terms and the number of DEGs in gynoparae (GP) vs. parthenogenetic females (PF). (B) GO terms and the number of DEGs in sexual females (SF) vs. GP. (C) GO terms and the number of DEGs in SF vs. PF. Up, up-regulated; Down, down-regulated. The term was considered to be enriched significantly when the *P* values of Pearson Chi-Square test were <0.05.

In the category of molecular function, DEGs of gynoparae vs. parthenogenetic females were significantly enriched in ion channel activity, oxidoreductase activity, nucelotidytransferase activity, DNA binding, protein dimerization activity, coenzyme binding, and cofactor binding (Fig. 4A). For sexual females vs. gynoparae, DEGs were primarily assigned to structural molecule activity, structural constituent of cuticle, catalytic activity, and chromatin binding (Fig. 4B). DEGs between sexual females and parthenogenetic females were significantly enriched in structural molecule activity, structural constituent of cuticle, chitin binding, carbohydrate binding, polysaccharide binding, DNA binding, coenzyme binding, protein dimerization activity, transcription regulatory activity, kinase activity, and ion channel activity (Fig. 4C).

**Figure 5:**
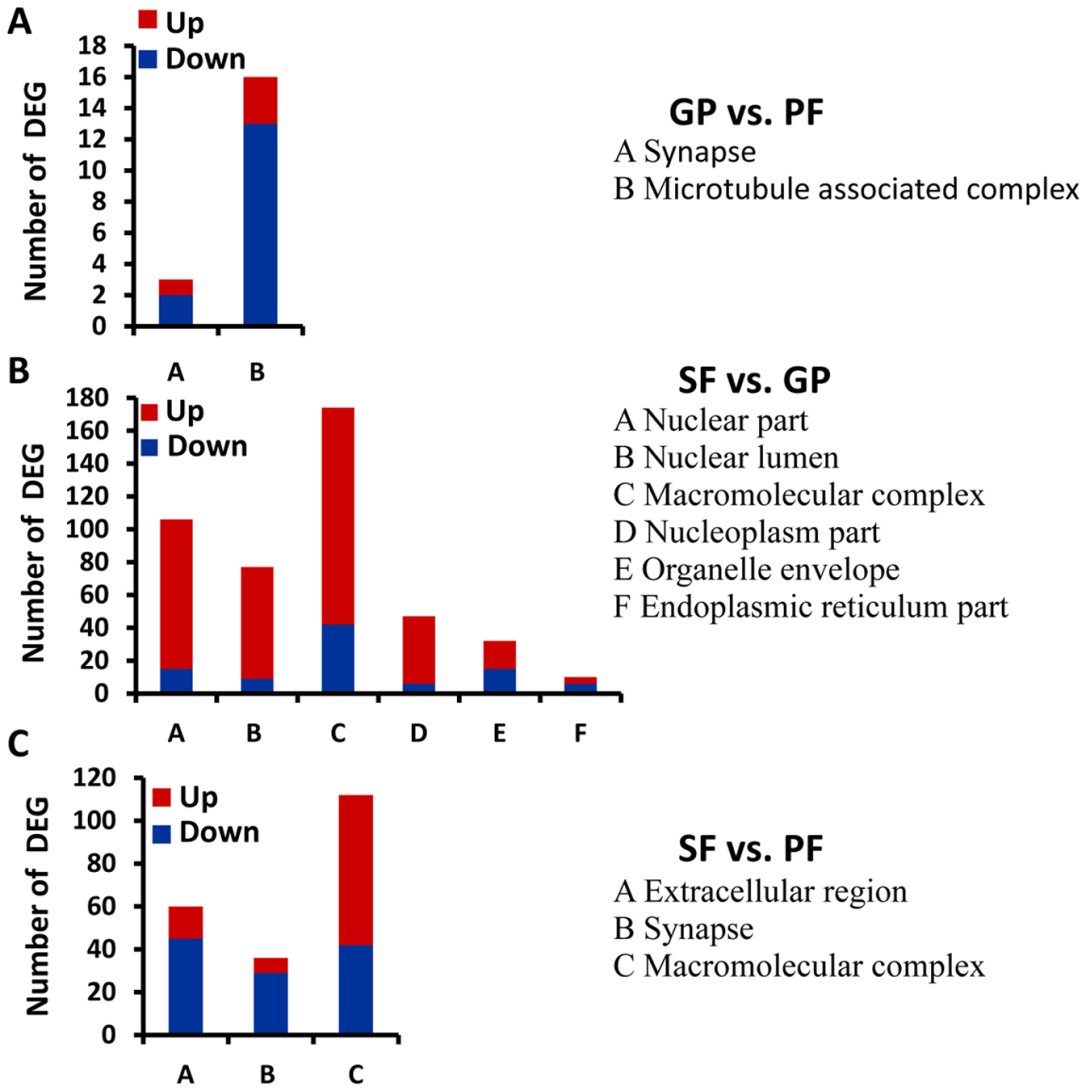
Significantly enriched GO terms of DEGs in the category of cellular component. (A) GO terms and the number of DEGs in gynoparae (GP) vs. parthenogenetic females (PF). (B) Represented GO terms and the number of DEG in sexual females (SF) vs. GP. (C) GO terms and the number of DEG in SF vs. PF. Up, up-regulated; Down, down-regulated. The term was considered to be enriched significantly when P values of Pearson Chi-Square test were <0.05.

In the category of cellular component, DEGs of gynoparae vs. parthenogenetic females were predominantly termed to synapse and microtubule associated complex (Fig. 5A). In the pair-wise comparison of sexual females and gynoparae, the most represented GO terms of DEGs were related to nuclear, macromolecular complex, organelle envelope, and endoplasmic reticulum part (Fig. 5B). For sexual females vs. parthenogenetic females, DEGs were significantly enriched in synapse, macromolecular complex, and extracellular region (Fig. 5C).

### Differentially regulated KEGG pathways among three reproductive morphs

**Figure 6:**
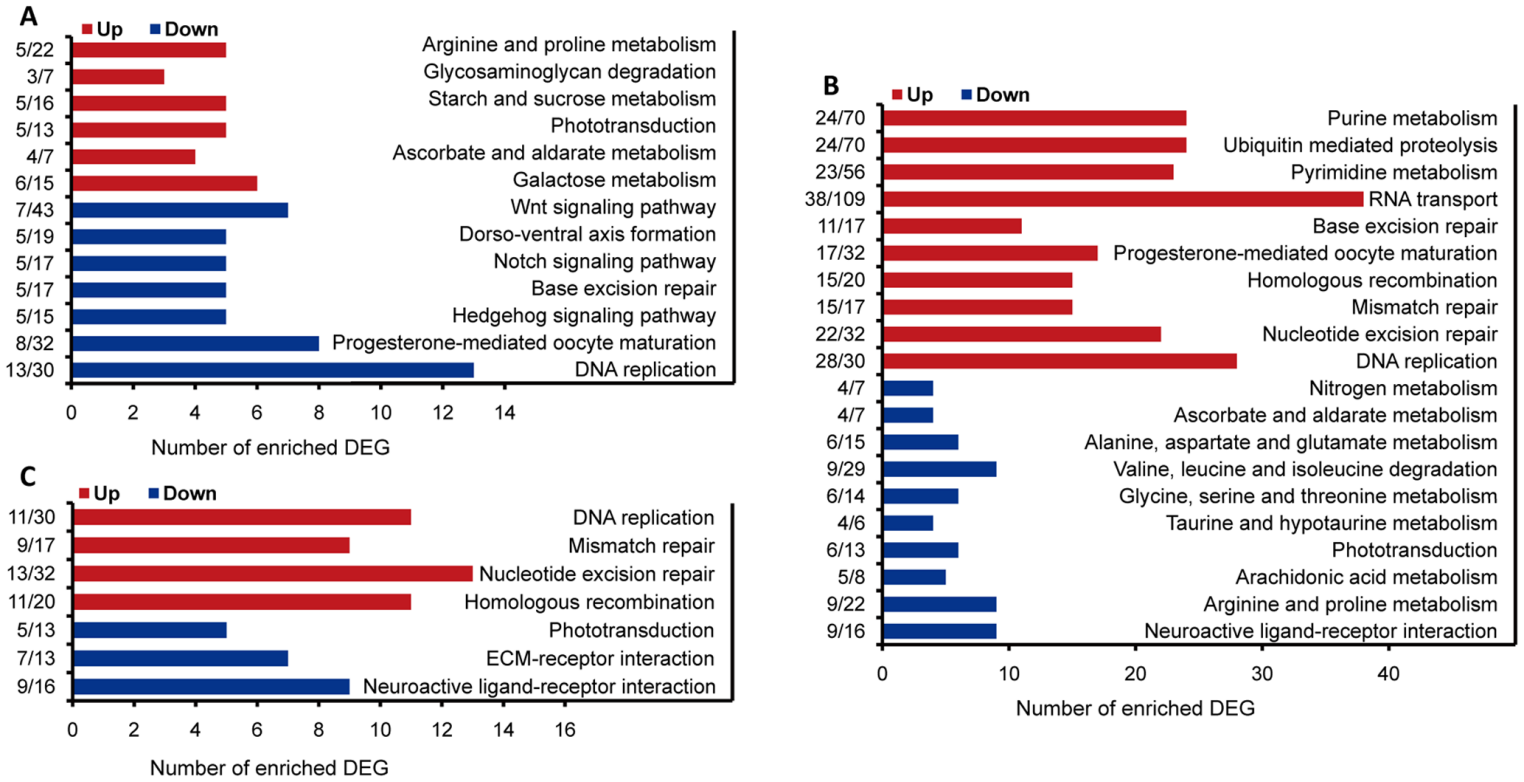
Significantly enriched KEGG pathways of DEGs in the pair-wise comparison of three reproductive morphs. (A) Significantly enriched pathways in gynoparae (GP) vs. parthenogenetic females (PF). (B) Significantly enriched pathways in sexual female (SF) vs. GP. (C) Significantly enriched pathways in SF vs. PF. KEGG enrichment analysis was performed separately with the up-regulated DEGs and the down-regulated DEGs. Up, up-regulated; Down, down-regulated. Numerators of the fractions indicate the number of DEGs in an enriched KEGG pathway, and denominators indicate the total number of genes in a KEGG pathway. Significantly enriched pathways were judged at *P*<0.05 in hypergeometric test.

To explore the pathways potentially involved in the switch of reproductive morphs and to distinguish the up- and down-regulated KEGG pathways, we performed KEGG enrichment analysis of up-regulated DEGs and down-regulated DEGs separately (see Table S5 for the list of significantly enriched KEGG pathways). Compared to parthenogenetic females, gynoparae had up-regulated phototransduction and metabolism of sugar and amino acids including galactose, glycosaminoglycan, ascorbate and aldarate, starch and sucrose, argininge and proline, while Hedgehog pathway, Notch and Wnt signaling, progesterone-mediated oocyte maturation, DNA replication, and dorso-ventral axis formation appeared to be down-regulated (Fig. 6A). In comparison to gynoparae, sexual females had up-regulated progesterone-mediated oocyte maturation, DNA replication, DNA repair, homologous recombination, ubiquitin mediated proteolysis, and metabolism of pyrimidine and purine. However, phototransduction, neuroactive ligand-receptor interaction, and metabolism of ascorbate and aldarate, arachidonic acid, Taurine and hypotaurine, nitrogen, and several amino acids were down-regulated in sexual females compared to gynoparae (Fig. 6B). Up-regulated DNA replication, DNA repair, homologous recombination as well as down-regulated neuroactive ligand-receptor interaction and phototransduction were also enriched in sexual females compared to parthenogenetic females (Fig. 6C).

### Validation of selected DEGs by RT-qPCR

To confirm the results of differentially expressed gene profiling, we selected 25 DEGs (Table 1), which are associated with phototransduction and neural signal transmission, cuticle composition, embryogenesis, and progesterone-mediated oocyte maturation for RT-qPCR validation. These enriched pathways have been proposed to be involved in the aphid reproductive mode switch [21]-[27]. Although the values appeared to vary, the expression of all DEGs tested in RT-qPCR had same trends to that in RNA-seq except the gene coding for mitogen-activated protein kinase kinase kinase 5 (*mapkkk5*) (Table 1). Spearman correlation by SPSS 11.0 software using log_2_ transformed data from RNA-seq and RT-qPCR showed a significant positive correlation between these two datasets (*r* = 0.921, *P*<0.001). The data indicate that our RNA-seq results are reliable and the DEGs identified in the RNA-seq analysis are potentially involved in the reproductive mode plasticity of cotton aphids.

**Table 1:** RT-qPCR validation.

Gene ID	Annotation	RNA-Seq		RT-qPCR	
		log_2_FC*	*P-*Value	log_2_FC*	*P-*Value
**GP vs. PF**					
Unigene2231	*arrestin*	1.901	0.000	2.966	0.039
Unigene26960	*ninaC*	1.790	0.000	2.430	0.000
Unigene27816	*Plc21C*	2.253	0.000	1.040	0.011
Unigene1473	*opsin* (*Uvop*)	3.358	0.000	3.751	0.000
Unigene11528	*period*	−4.812	0.000	−3.436	0.000
Unigene24328	*cuticlin-1*	−6.319	0.000	−5.471	0.000
Unigene26518	*chitinase 3*	−5.288	0.000	−5.290	0.000
Unigene1224	*Dat*	1.103	0.002	1.066	0.004
Unigene23404	*Neurogenic locus Notch*	−3.946	0.000	−1.803	0.000
Unigene16031	*Wnt-2*	−3.211	0.000	−2.023	0.001
Unigene11468	*cyclin-D2*	−2.572	0.001	−1.054	0.007
**SF vs. GP**					
Unigene2231	*arrestin*	−3.484	0.000	−3.804	0.031
Unigene1224	*Dat*	−2.337	0.000	−0.822	0.012
Unigene24828	*GABA-B receptor 1*	−2.031	0.000	−2.244	0.005
Unigene2776	*CDK-activating kinase assembly factor MAT1*	4.254	0.000	4.549	0.018
Unigene2846	*cyclin-B*	6.134	0.000	5.367	0.063
Unigene19255	*bub1b*	4.662	0.000	0.349	0.241
Unigene10663	*mapkkk5*	−6.111	0.000	0.511	0.047
Unigene4937	*APC subunit 10*	3.374	0.000	4.324	0.000
CL420.Contig2	*Male-specific lethal 1 homolog* (*MSL1*)	2.128	0.009	2.258	0.037
Unigene17152	*Hunchback*	6.057	0.000	5.204	0.000
Unigene28333	*apterous*	3.628	0.000	2.898	0.000
Unigene4941	*takeout*	7.318	0.000	3.095	0.000
**SF vs. PF**					
Unigene11528	*period*	−6.503	0.000	−6.062	0.000
Unigene17798	*clock-controlled gene 3*	−4.764	0.000	−3.291	0.000
Unigene4941	*takeout*	7.682	0.000	4.434	0.000
Unigene24381	*collagen*	−8.221	0.000	−6.229	0.000
Unigene26518	*chitinase 3*	−8.257	0.000	−6.789	0.000
Unigene14334	*keratin*	−7.796	0.000	−4.104	0.000
Unigene2846	*cyclin-B*	2.171	0.000	3.299	0.081
Unigene27050	*vitellogenin*	2.715	0.000	2.521	0.000
Unigene28333	*apterous*	3.158	0.000	3.761	0.000

*FC, fold change. The results represent the mean of tetraplicate experiments (n = 4).

### DEGs related to phototransduction, cuticle composition, progesterone-mediated oocyte maturation, and endocrine regulation

The transformation of cotton aphid reproductive morphs is triggered by shortening photoperiod. The photoperiod change is recognized by the protocerebrum in insect brain through the cuticular head capsules and putative photoreceptors located in the compound eyes [21]. In our RNA-seq analysis, 5 photoperiod-related genes including *neither inactivation nor afterpotential C* (*ninaC*), *Phospholipase C at 21C* (*Plc21C*), *transient receptor potential-like* (*trpl*), *guanine nucleotide-binding protein subunit beta-2* (*Gnb2*) and *arrestin* were found to be differentially expressed among the 3 reproductive morphs and enriched in GO category and KEGG pathway. These genes are not reported for the pea aphid. The transcript levels of *ninaC*, *Plc21C*, *trpl*, *Gnb2* and *arrestin* were increased 4.3, 4.8, 2.4, 4.0 and 3.7 fold, respectively in gynoparae relative to parthenogenetic females. However, the mRNA levels of *ninaC*, *Plc21C*, *trpl*, *Gnb2* and *arrestin* were decreased 19.0, 6.6, 10.5, 14.2 and 11.2 fold, respectively in sexual females in comparison to gynoparae. When compared to parthenogenetic females, sexual females had 6.0-, 4.4-, 3.5- and 3.0-fold lower levels of *ninaC*, *trpl*, *Gnb2* and *arrestin* expression respectively, whereas levels of *Plc21C* had no significant change between these two morphs. The data suggest that these genes are likely to be involved in reproductive mode plasticity of the cotton aphid, but further studies are needed to elucidate why these genes showed converse expression patterns between the transition from the parthenogenetic female to gynopara and from the gynopara to sexual female, and how these genes function in reproductive mode switch.

Cuticles of three reproductive morphs are apparently different in color and sclerotization. Gynoparae are black and with hard cuticles. Bodies of parthenogenetic females are yellow in majority and soft, whereas sexual females have dark green soft bodies. Our RNA-Seq libraries yielded DEGs coding for 36 cuticle proteins (CPs), including 7 CPs with RR-1 domain, 20 CPs with RR-2 and 9 CPs lacking RR domain [22]. When gynoparae were compared to parthenogenetic females, 21 differentially expressed *CP* genes including 1 with RR-1, 14 with RR-2 and 6 without RR domain were identified and showed down-regulated (−10.7 to −62.1 fold). From gynoparae to sexual females, 4 RR-1 and 5 RR-2 *CP* genes appeared to be down-regulated (−2.0 to −29.4 fold), whereas 1 RR-2 *CP* was up-regulated for 4.1-fold. In the pea aphid, the microarray analysis detects altered expression of 19 *CP* genes between the parthenogenesis and sexual reproduction, including 10 down-regulated (FC  = −1.7 to −2.7) transcripts with RR domain and 9 up-regulated (FC  = 1.8 to 2.3) transcripts without RR domain [6], [23]. Thus, our data broaden the view of candidate *CP* genes in reproductive morph transition of aphids. The result suggests that different aphid species have common and divergent features of *CP* genes for their respective traits of reproductive morphs. As cuticle proteins have variable functions and are in network with Chitin and other factors, the role of CPs in cuticular phenotypes and reproductive polyphenism needs further investigation. Notably, the expression levels of gene coding for Dopamine N-acetyltransferase (*Dat*), which plays a key role in sclerotization [21], was increased 4.7-fold in gynoparae but further declined 9.4-fold in sexual females, suggesting that *Dat* may be required for the body sclerotization of gynoparae.

The cotton aphid has two symmetric ovaries, each of which has approximately 6 ovarioles. Sexual females undergo sexual reproduction and oviposition, whereas gynoparae and parthenogenetic females reproduce in parthenogenesis. However, in gynoparae, only the primary embryos are able to develop maturely. In the present study, we found that genes related to the progesterone-mediated oocyte maturation pathway, including *cyclin-dependent kinase 2* (*cdk2*), *polo-like kinase 1* (*plk1*), *cyclin-B*, *cytoplasmic polyadenylation element binding protein 1* (*cpeb1*), *cyclin-D2*, *mitotic checkpoint serine/threonine-protein kinase beta* (*bub1b*), *cell division cycle 2* (*cdc2*) and *mitotic spindle assembly checkpoint protein* (*mad2*), were down-regulated in gynoparae but up-regulated in sexual females. The expression levels of *cdk2*, *plk1*, *cyclin-B*, *cpeb1*, *cyclin-D2*, *bub1b*, *cdc2* and *mad2* were declined 5.8, 16.9, 15.6, 8.5, 27.1, 8.4, 10.9 and 10.3 fold, respectively in gynoparae compared to parthenogenetic females, whereas their levels were elevated 12.2, 26.0, 70.2, 7.9, 95.3, 25.3, 18.9 and 36.8 fold from gynoparae to sexual females. In comparison to parthenogenetic females, these genes also showed up-regulated in sexual females but at lower levels. The data suggest that the progesterone-mediated oocyte maturation pathway may be important in the regulation of oogenesis and embryogenesis in reproductive morphs of the cotton aphid. In the pea aphid, which has no gynopara form, the final determination of reproductive mode depends upon the fate of pre-oocyte in germarium. The haploid oocyte through normal meiosis of pre-oocyte develops to an egg, whereas the diploid oocyte through modified meiosis II of pre-oocyte enters into embryonic development [9], [24], [25]. It has been shown that cell cycle and meiosis related genes, including *cyclin-J*, *cytoplasmic linker associated protein 1* (*clasp1*), *dipeptidase 1* (*Dip-1*), *nuclear distribution E homolog-like 1 nudel*), *oo18 RNA-binding protein* (*orb*), *bicaudal-C*, *kelch*, *nanos-like protein nanos*), *orthodenticle*, *hunchback* and *caudal* are expressed differently between the sexually and asexually produced oocytes and embryos of the pea aphid [25]–[27]. Thus, the cotton aphid and pea aphid share common cell cycle regulation in reproductive mode transition but may diverge in specific regulatory mechanisms.

Our DEG profiling showed that the transcript levels of gene coding for juvenile hormone acid methyltransferase, a key enzyme in juvenile hormone (JH) synthesis, were significantly decreased 1.7-fold in gynoparae compared to parthenogenetic females, 2.8-fold in sexual females relative to gynoparae, and 4.8-fold in sexual females compared to parthenogenetic females, respectively. Interestingly, the mRNA levels of genes coding for allatostatin, which inhibits JH synthesis [28] and JH esterase, which hydrolyzes JH [29] were increased 1.6- and 2.1-fold, respectively in gynoparae compared to parthenogenetic females. Decreased JH synthesis and increased JH hydrolysis are likely to retain the lower levels of JH titer in gynoparae, as seen in sexuparae of the pea aphid [30], [31]. Application of a JH analog to gynoparae of the green peach aphid, *Myzus persicae* and the black bean aphid, *Aphis fabae* leads to the production of parthenogenetic aphids instead of sexual progenies [32]. Similarly, ectopic treatment of JH on sexuparae of the pea aphid causes the presence of ovaries with both viviparous and oviparous ovarioles [33]. In this study, the transcript levels of *Krüpple homolog-1*, an early JH response gene [34] was also declined 2.3-fold in gynoparae compared to parthenogenetic females. Taken together, these data suggest that JH action in reproductive mode switch may be conserved in the cotton aphid.

## Conclusions

The present study represents the first report on a large-scale analysis of differentially expressed genes among reproductive morphs of the cotton aphid. Our data demonstrated the altered gene expression in phototransduction, cuticle modification and oogenesis/embryogenesis, which confirms the previous reports on the pea aphid via the microarray analysis. However, our result showed divergent involvement of molecules in these pathways. In addition, our study added a number of genes to the list of candidate genes involved in the reproductive mode plasticity of aphids. Thus, this result provides a comprehensive gene expression profile among reproduction morphs of a host-alternating aphid species, and extends our knowledge of the putative regulatory cascades in aphid reproductive polyphenism. Further studies on the candidate genes will help to unfold the molecular mechanisms underlying the transition from parthenogenesis to sexual reproduction of the cotton aphid as well as to develop novel strategies for aphid control.

## Supporting Information

Figure S1 **The holocyclic life cycle of the cotton aphid.** The overwintering egg hatches and develops to fundatrix on the primary host plants in spring. The fundatrix reproduces in parthenogenesis for fundatrigena, which undergoes 2–3 generations on the primary hosts and produce alate parthenogenetic females. Alate adults migrate to the second hosts such as the cotton in late spring. During the summer, the parthenogenetic females (PF) reproduce in parthenogenesis over a dozen of generations in the cotton field. In the fall, the alate gynoparae (GP) and sexual males (SM) are reproduced and migrate to the primary hosts. The gynoparae produce sexual females (SF) which oviposit fertilized eggs after mating with the males. Scale bars, 0.2 mm.
(TIF)

Figure S2 **Overview of DEG identification among three reproduction morphs of cotton aphids.** Total clean reads of each library were obtained by filtering out the adapter sequences and low-quality reads from the raw reads. The unique clean reads and unigene sequences were acquired by mapping to the cotton aphid transcriptome database. The genes identified in the merged datasets of 9 libraries were further analyzed by filtering out the repetitive and unannotated genes. Fold change ≥2 and *P*<0.01 were used to determine differentially expressed genes (DEGs) in the pair-wise comparison of three reproductive morphs. PF, parthenogenetic females; GP, gynoparae; sexual females.
(TIF)

Figure S3 **Gene ontology analysis of DEGs in the pair-wise comparison of three reproductive morphs.** PF, parthenogenetic females; GP, gynoparae; SF, sexual females.
(TIF)

Table S1 **Primers used in RT-qPCR.**
(DOCX)

Table S2 **Pearson correlation coefficient analysis among three replicates of each reproductive morph.**
(DOCX)

Table S3 **The DEGs in the pair-wise comparison of three reproductive morphs.** Relative gene expression levels in each reproductive morph were calculated by average RPKM of 3 replicates. Differentially expressed genes (DEGs) were determined by the cutoff criteria of fold change ≥2 and *P*<0.01. PF, parthenogenetic females; GP, gynoparae; SF, sexual females.
(XLSX)

Table S4 **Significantly enriched GO terms in the pair-wise comparison of three reproductive morphs.** No. of DEGs indicate the number of differentially expressed genes in a GO term, whereas No. of genes represent the total number of orthologous genes in a GO term. PF, parthenogenetic females; GP, gynoparae; SF, sexual females.
(XLSX)

Table S5 **Significantly enriched KEGG pathways in the pair-wise comparison of three reproductive morphs.** KEGG enrichment analysis was performed with the up-regulated DEGs and the down-regulated DEGs separately. No. of DEGs, the number of differentially expressed genes in a KEGG pathway; No. of genes, the total number of orthologous genes in a KEGG pathway. PF, parthenogenetic females; GP, gynoparae; SF, sexual females.
(XLSX)
