# Supplementary figures and images for: Comparative Transcriptional Analysis of Asexual and Sexual Morphs Reveals Possible Mechanisms in Reproductive Polyphenism of the Cotton Aphid
Source: PLoS One. 2014 Jun 10;9(6):e99506. doi: 10.1371/journal.pone.0099506 (PMC4051768; doi:10.1371/journal.pone.0099506)

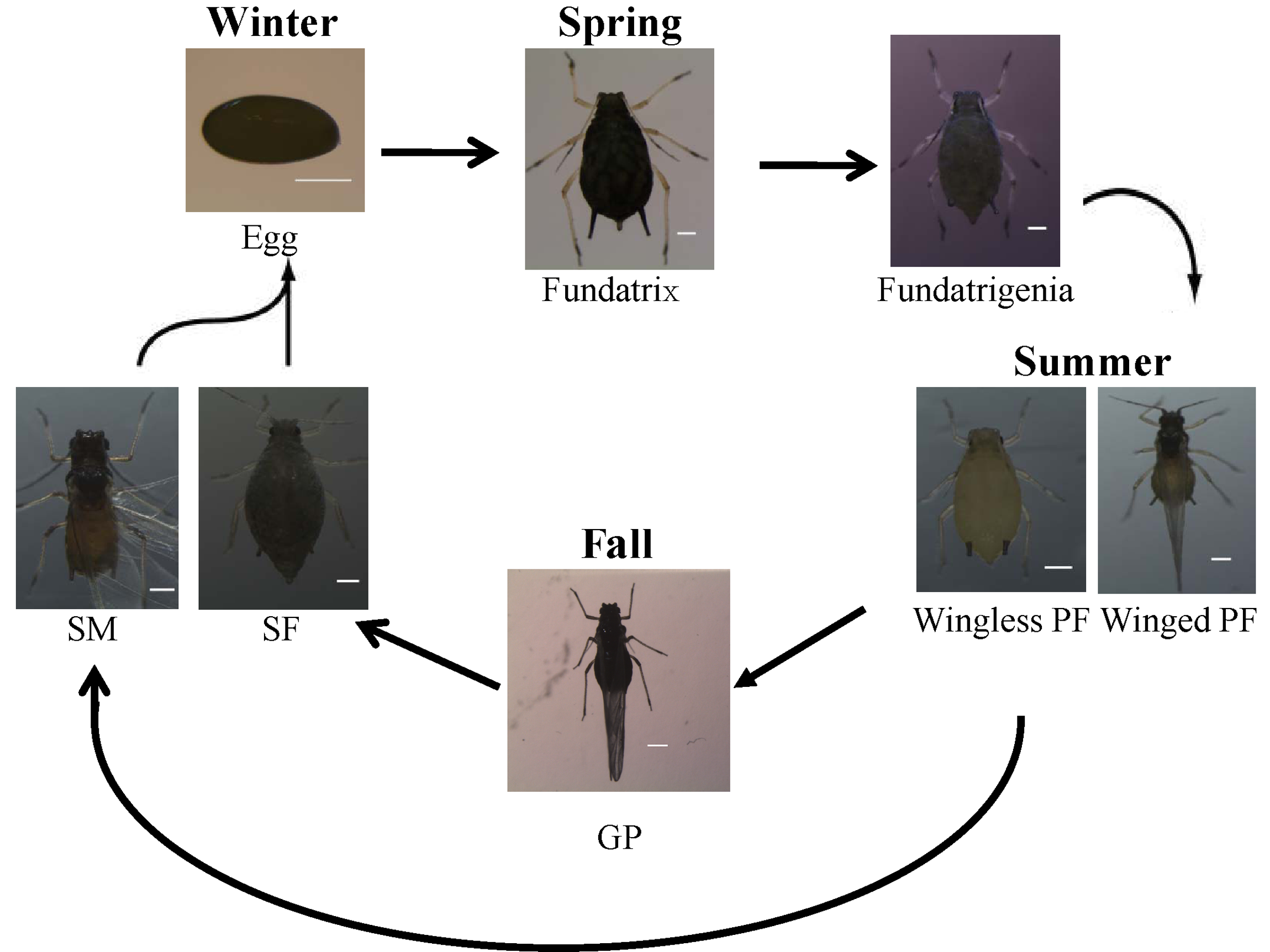

Supplement: Figure S1 — The holocyclic life cycle of the cotton aphid. The overwintering egg hatches and develops to fundatrix on the primary host plants in spring. The fundatrix reproduces in parthenogenesis for fundatrigena, which undergoes 2–3 generations on the primary hosts and produce alate parthenogenetic females. Alate adults migrate to the second hosts such as the cotton in late spring. During the summer, the parthenogenetic females (PF) reproduce in parthenogenesis over a dozen of generations in the cotton field. In the fall, the alate gynoparae (GP) and sexual males (SM) are reproduced and migrate to the primary hosts. The gynoparae produce sexual females (SF) which oviposit fertilized eggs after mating with the males. Scale bars, 0.2 mm. (TIF) [file pone.0099506.s001.tif]

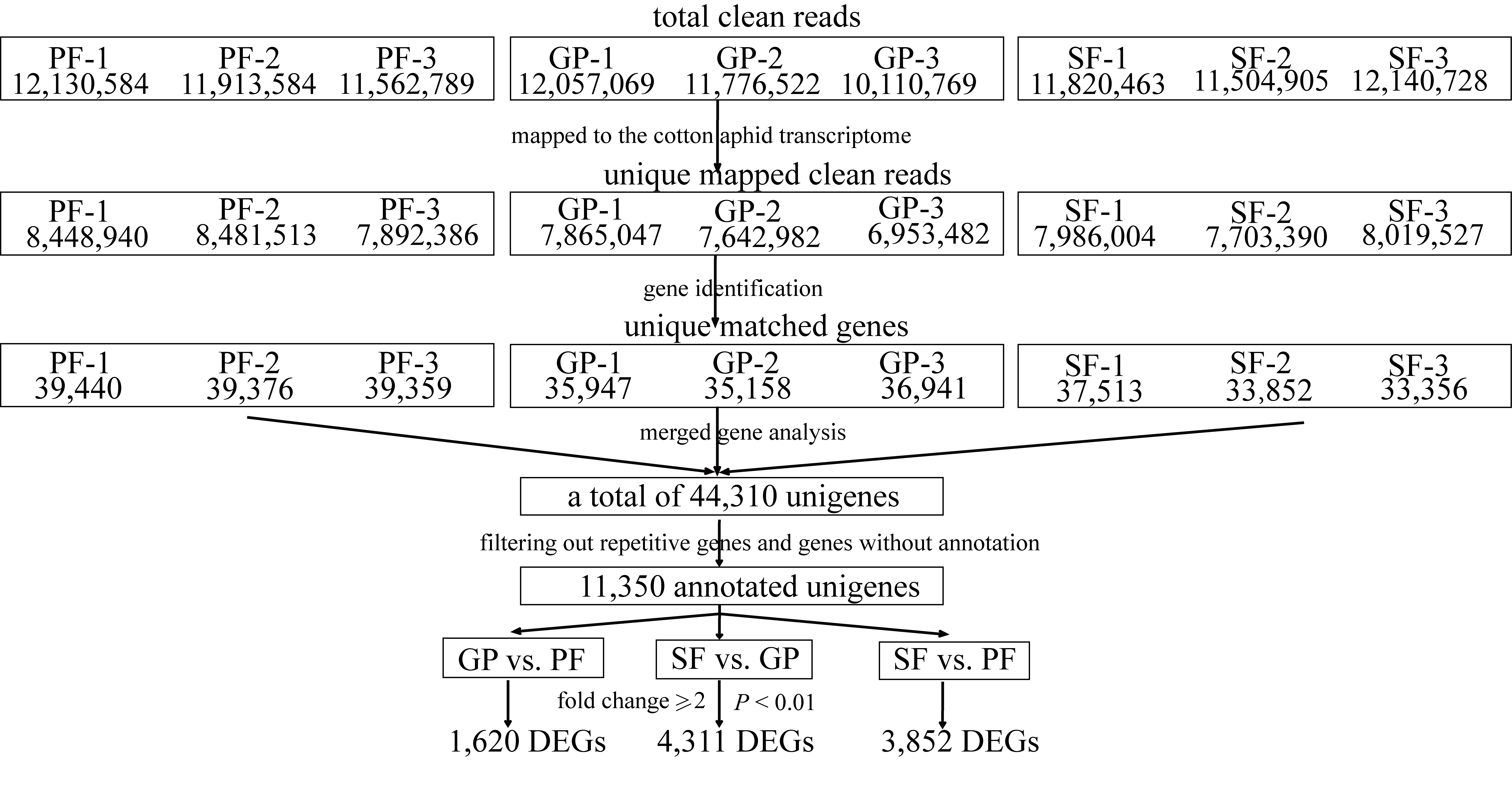

Supplement: Figure S2 — Overview of DEG identification among three reproduction morphs of cotton aphids. Total clean reads of each library were obtained by filtering out the adapter sequences and low-quality reads from the raw reads. The unique clean reads and unigene sequences were acquired by mapping to the cotton aphid transcriptome database. The genes identified in the merged datasets of 9 libraries were further analyzed by filtering out the repetitive and unannotated genes. Fold change ≥2 and P<0.01 were used to determine differentially expressed genes (DEGs) in the pair-wise comparison of three reproductive morphs. PF, parthenogenetic females; GP, gynoparae; sexual females. (TIF) [file pone.0099506.s002.tif]

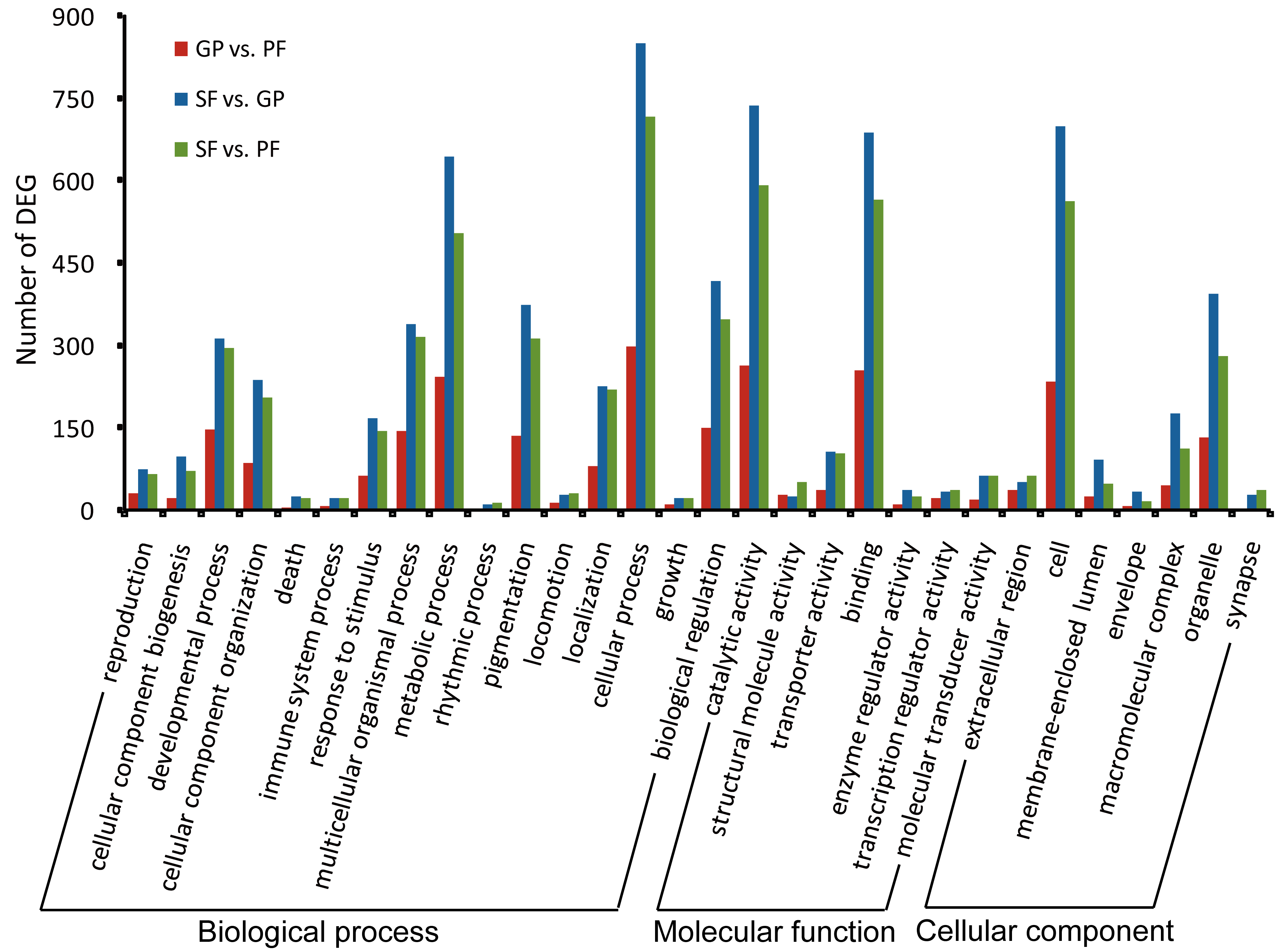

Supplement: Figure S3 — Gene ontology analysis of DEGs in the pair-wise comparison of three reproductive morphs. PF, parthenogenetic females; GP, gynoparae; SF, sexual females. (TIF) [file pone.0099506.s003.tif]
