# Supplementary material for: Comparative Transcriptional Analysis of Asexual and Sexual Morphs Reveals Possible Mechanisms in Reproductive Polyphenism of the Cotton Aphid
Source: PLoS One. 2014 Jun 10;9(6):e99506. doi: 10.1371/journal.pone.0099506 (PMC4051768; doi:10.1371/journal.pone.0099506)
Supplement: Table S1 — Primers used in RT-qPCR. (DOCX) [file pone.0099506.s004.docx]

**Table S1. Primers used in RT-qPCR.**

| **Gene** | **Forward (5'-3')** | **Reverse (5'-3')** |
| --- | --- | --- |
| *arrestin* | CAGCCAGGTGACGATGATAATGGAA | ACCTCTTGTTGATGGAGCGTATTGT |
| *ninaC* | TCCGACGATGACGATAGCGAAGT | GTGCCGTTGACCACAACACAGTA |
| *Plc21C* | AAGCCTTGACTTCGTGTGGATTACC | GCACAACTTCAGGTCGAGTCGTAAG |
| *opsin* (*Uvop*) | GCAAATGCTCAATCAGCGGAAGTT | CCAATCATAGCGACCACTGCGTAT |
| *period* | CGGTTACGGTTACGGTTCGAGTTAC | CGCCACCTCCTTGATAATGGTCTTG |
| *cuticlin-1* | CCTGCTGGTGTCCTGTCTGGTAT | CGACTTGTGCATCGTCTGTGGTT |
| *chitinase 3* | GCGTGCGTTGTAGTCACAAGAGTA | GCTGCCAATATCGTAGCTGGTAGT |
| *Dat* | CTAAAGCGGCAGAGAGACTTGGTTT | TGAAGAATCGTGAGGAGGAGGAGTT |
| *Neurogenic locus Notch* | GTGCATCGACGGTATCAACAAGT | AACGCCAACCAGCCACTAGG |
| *Wnt-2* | CAGACGACGAAGAGCCGAAGA | TTGGTCCTGGTGAACTGGTGAG |
| *cyclin-D2* | GCTCTTCTCGGACGGTGTCTT | CGTAGCGGTAGCACTTATTGTCAC |
| *GABA-B receptor1* | GATAGCGAGTGTGATCCTGGACTTG | TTCGGCAACAGTTGTGCATACAGTA |
| *MAT1* | CAACGTGTGCGGGCATACCAT | CCATTACGCCGTAGAGGAATGAGAC |
| *cyclin-B* | ATTGGTGCCACAAGTCTACTGCTG | CATCCTTGGTGAAGGCATTGTCTGA |
| *bub1b* | TTGTTGGTAGTTCGCCGTTATCCG | GCACGAGCAGCCTCAAGTCAA |
| *mapkkk5* | ATGGCTCCTGAAGTGATGAACTGC | TAGTAAGTAGCCGCTGCTCCAAGT |
| *APC subunit 10* | TGGTAGTCAAGCTATATGGAGTCT | TATATCACGCACAACTGTCTTACG |
| *MSL1* | CGTGCGTTGATGGTCATTGTTAGC | CGCCGTTTACAATCCGACACTCTT |
| *hunchback* | CTGTGCCAGTGGTGATGGTTCC | TGCTGCTGTTGCTGATGATGATGAT |
| *apterous* | CACCATTACCAACAGTTCGGACCTT | TGGACGAAGGATACGGCGACTG |
| *takeout* | TGAAGCTAATGTCCTCGTCCAATGT | TCGGTCATGCAAGCATTTACCTCAG |
| *clock-controlled gene 3* | AGTGTTGTTAGTGCTTCCTGCTA | CCTATTGTTGTTGACTCTGGTGAC |
| *collagen* | TTTGGTCCCTTGCTATGTCCTTGTG | ACTAACTGGTGAACGAGGTCCTGAT |
| *keratin* | AGTTCACCATTACGAACACCACGAT | CGAGTCATCTCCGCCACTACCA |
| *vitellogenin* | AGACTACGAGGACCCAATGAGAGG | CAGACGATGTGCCGAACGAGTG |
| *rpl27* | GAAGGTACAGCGGAAAGACAATATG | CTGTGACATGCGTTTGGTGAC |
