# Supplementary material for: Comparative Transcriptional Analysis of Asexual and Sexual Morphs Reveals Possible Mechanisms in Reproductive Polyphenism of the Cotton Aphid
Source: PLoS One. 2014 Jun 10;9(6):e99506. doi: 10.1371/journal.pone.0099506 (PMC4051768; doi:10.1371/journal.pone.0099506)
Supplement: Table S2 — Pearson correlation coefficient analysis among three replicates of each reproductive morph. (DOCX) [file pone.0099506.s005.docx]

**Table S2. Pearson correlation coefficient analysis among three replicates of each reproductive morph**

| **Parthenogenetic female (PF)** | | | | | |
| --- | --- | --- | --- | --- | --- |
| ***r*** | PF-2 | PF-3 | ***P*** | PF-2 | PF-3 |
| PF-1 | 0.569 | 0.715 | PF-1 | 0.000E00 | 0.000E00 |
| PF-2 |  | 0.76 | PF-2 |  | 0.000E00 |
| **Gynopara (GP)** | | | | | |
| ***r*** | GP-2 | GP-3 | ***P*** | GP-2 | GP-3 |
| GP-1 | 0.530 | 0.617 | GP-1 | 0.000E00 | 0.000E00 |
| GP-2 |  | 0.964 | GP-2 |  | 0.000E00 |
| **Sexual female (SF)** | | | | | |
| ***r*** | SF-2 | SF-3 | ***P*** | SF-2 | SF-3 |
| SF-1 | 0.916 | 0.862 | SF-1 | 0.000E00 | 0.000E00 |
| SF-2 |  | 0.982 | SF-2 |  | 0.000E00 |
